# The Limitations of Dentistry

**Published:** 1921-09-24

**Authors:** 


### THE LIMITATIONS OF DENTISTRY.

Public fancy is always attracted by comparisons of present-day progress with the methods of our forefathers. But though it is true that we have remarkable improvements in almost every sphere of professional and industrial life, yet how many of the general public take full advantage of these things—even though they know them to exist?

For this reason we hope that a considerable section of the public—sufficient at least to spread the news—has visited the International Dental Exhibition which was opened last week. It is only recently that an Act of Parliament made it practically impossible for anyone in future to enter the true dental profession without first passing through satisfactory training and examinations. Therefore one can assume that in dentistry a higher standard of personal efficiency may soon be expected. A mere glance round the recent exhibition will have shown that of improved mechanical efficiency there is already abundance, and in future, by a combination and extension of the two, will disappear the layman's last vestige of excuse for not having reasonably sound teeth.

As a contemporary wisely remarks, men may fight on their stomachs, but they live to a great extent on their teeth, and when these deteriorate and decay the ill-effect upon health is both obvious and immediate. By allowing their dentition to fall to such extreme deficiency the greater number of our adult population run absurdly unnecessary risks. There is, indeed, the most urgent call for further public education upon the simplicity, delicacy, and precision with which the commoner dental troubles can be controlled.

On the other hand, there is an important point to be remembered by those who would do this teaching. Bad teeth may be either the result or the cause of disease—or, indeed, both. In the overwhelming proportion of cases dental decay begins in the first quarter of life. Sometimes it is due to direct neglect of the teeth, but, as in the recent researches on vitamines, etc., the essential cause may also lie in some diseased or deficient condition of the body as a whole.

In such circumstances all the array of instruments and mechanical wonders seen at the Horticultural Hall, be they placed in even the most skilful hands, will not save the situation. It is the underlying cause which must be treated, and our children, when their teeth decay, must be examined most carefully for essential defects in their general health. The constitution of a single tooth, just as the constitution of the whole body, may be undermined and permit, within it, the destructive activities of bacteria.

In commenting on this matter, *The Times* most justifiably protests that "the value of good dentistry has occasionally been disparaged because cures were not obtained by means of it. Dentistry can alleviate only those afflictions which are due to defects of the teeth; it cannot perform the miracle of eradicating a general disease of the body."

